# Risk Factors of Financial Exploitation Versus Scam

**DOI:** 10.1093/geroni/igab046.337

**Published:** 2021-12-17

**Authors:** Pi-Ju Liu, Stacey Wood, Aining Wang, Yaniv Hanoch, David Hengerer, Mary Muskat

**Affiliations:** 1 Purdue University, West Lafayette, Indiana, United States; 2 Scripps College, Claremont, California, United States; 3 University of Southampton, Southampton, England, United Kingdom; 4 Claremont Graduate University, Claremont, California, United States; 5 Purdue University, West Lafayette, California, United States

## Abstract

Financial exploitation (FE) perpetrators are usually seen in a position of trust, such as family members or friends, whereas perpetrators of scam tend to be unknown individuals. Few empirical studies have examined victim risk factors, and this study aimed to systematically compare risk factors of FE versus scam. One-hundred-and-ninety-five adults (ages 18-89) were recruited to complete a 60-minute survey and interview at Purdue University in Indiana (n1=97) and Scripps College in California (n2=98). Risk factors assessed included cognitive tasks (overall cognition, memory, and executive decision), socio-emotional questionnaires (depression, resilience, ostracism, and social integration), financial measures (numeracy, objective financial knowledge, retirement worries, and financial well-being), physical health and demographics (age, gender, education level, marital status, ethnicity). Additionally, participants reported experiences of FE and scam, including (1) the 11-item short-form Older Adult Financial Exploitation Measure, (2) seven questions on scam from the Health and Retirement Study, and (3) likelihood to contact a scammer after reviewing lottery scam materials. The three dependent variables were log-transformed before OLS regression models were built. Each dependent variable was associated with different risk factors. Lower standard of living (p=.02) and ostracism (p<.05) independently predicted FE. Lower physical health (b=-.02, p=.003) was the strongest predictor of scam, with lower level of financial well-being (p=.02) serving as an independent predictor. For lottery scams contact likelihood, ostracism (b=.04, p=.005) and being male (b=-.23, p=.04) were the strongest predictors. Since risk factors differed between FE and scam, prevention and intervention programs should target the unique profiles of risk factors for each.

